# A successful case of abdominal aortic aneurysm complicated by abdominal aortocaval fistula by endovascular aneurysm repair

**DOI:** 10.1016/j.ijscr.2025.111806

**Published:** 2025-08-17

**Authors:** Kosuke Tsumura, Masateru Uchiyama

**Affiliations:** a Kawasaki Aortic Center, Kawasaki Saiwai Hospital, 31-27, Omiya-cho, Sakae-ku, Kawasaki, Kanagawa 212-0014, Japan; b Department of Cardiovascular Surgery, Teikyo University, 2-11-1, Kaga, Itabashi-ku, Tokyo 173-8605, Japan

**Keywords:** Abdominal aortic aneurysm, Rupture, Aortocaval fistula, EVAR, Case report

## Abstract

**Introduction:**

An aortocaval fistula (ACF) associated with an abdominal aortic aneurysm (AAA) is relatively rare and causes various clinical symptoms. No consensus has been reached regarding surgical treatment for high-risk patients.

**Presentation of case:**

We report the case of a 76-year-old male with an AAA-inferior vena cava (IVC) fistula, incidentally diagnosed during examination for chronic heart failure and renal dysfunction. Contrast-enhanced computed tomography (CT) revealed IVC compression by a saccular AAA (56.6 mm × 73.7 mm), contrast agent inflow into the IVC, and a large fistula. The patient underwent emergency endovascular aneurysm repair (EVAR) to prevent rupture and reduce shunt volume. The postoperative course was uneventful. Follow-up CT showed shrinkage of the AAA diameter despite a slight residual shunt into the IVC.

**Discussion:**

ACF is a relatively rare comorbidity of AAA. The standard surgical intervention for AAA complicated by ACF involves abdominal aortic graft replacement and fistula closure. However, no consensus exists regarding the optimal surgical approach for high-risk patients. Our surgical intervention was selected based on the patient's condition, as well as the ACF and AAA characteristics. Considering the potential perioperative risks associated with renal dysfunction and chronic heart failure, we opted to perform EVAR. EVAR allowed for a safe and reliable procedure for managing AAA complicated by ACF, improving the patient's condition and preventing rupture.

**Conclusion:**

This case provides valuable insight into the successful emergency EVAR treatment for a high-risk patient experiencing chronic heart failure and acute kidney injury due to increased preload from an ACF.

## Highlights

• Aortocaval fistula (ACF) is rare comorbidity of abdominal aortic aneurysm (AAA).
• ACF causes various clinical symptoms which complicate preoperative diagnosis.
• No consensus exists on the optimal surgery for high-risk AAA patients with ACF.
• Endovascular aneurysm repair is a reliable procedure for AAA complicated by ACF.

## 1 Introduction

Aortocaval fistula (ACF) associated with abdominal aortic and iliac artery aneurysms is relatively rare and is thought to cause various clinical symptoms. These symptoms include heart failure (due to preload associated with shunts), oedema, and deep vein thrombosis (due to impaired venous return associated with venous hypertension) [[1], [2], [3], [4]]. Conventionally, open abdominal aortic replacement has been the prevailing surgical option for abdominal aortic aneurysms (AAA) with ACF. Recent reports indicated good surgical outcomes with endovascular aneurysm repair (EVAR) [4,5]. However, no consensus has been reached regarding surgical treatment for high-risk patients. We report a case involving successful surgical treatment for an AAA complicated by an abdominal ACF, which presented with heart failure and impaired renal function, using EVAR without any complications. This work has been reported in line with the SCARE 2025 criteria [6].

## 2 Case report

**Fig. 1:**
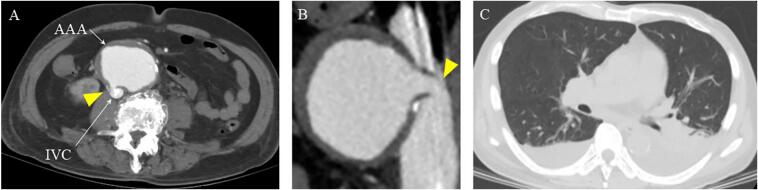
Preoperative Computed Tomography. (A, B) Large aortocaval fistula was observed and the inferior vena cava was contrasted in the arterial phase (shown as yellow arrows). (C) Bilateral chest pleural effusion. Abbreviations: AAA, abdominal aortic aneurysm; IVC, inferior vena cava. (For interpretation of the references to colour in this figure legend, the reader is referred to the web version of this article.)

A 76-year-old male presented with dyspnoea on exertion and was diagnosed with exacerbated chronic heart failure at another institution. Despite oral medication, heamodialysis was performed due to worsening oedema, oliguria, and dyspnoea at rest. During hospitalisation, contrast-enhanced computed tomography (CT) revealed an incidental diagnosis of AAA-inferior vena cava (IVC) fistula, and the patient was referred to our institution. The patient had a medical history of chronic heart failure, diabetes, dyslipidaemia, and chronic obstructive pulmonary disease. Initial physical examination findings showed stable hemodynamic values but coarse crackles and bilateral leg oedema. Laboratory findings upon admission revealed mild liver dysfunction (aspartate aminotransferase, 70 IU/L; alanine aminotransferase, 106 IU/L), renal dysfunction (creatinine, 2.16 mg/dL), and elevated cardiac marker levels (brain natriuretic peptide, 1175 pg/mL). Initial contrast-enhanced CT revealed compression of the IVC by a saccular AAA (56.6 mm × 73.7 mm), contrast agent inflow into the IVC, a large fistula, and bilateral chest pleural effusion (Fig. 1A-C). Transthoracic echocardiography showed preserved left ventricular systolic function with an ejection fraction of 56 % and normal valve function, but apical hypokinesis and IVC enlargement (22 mm). Considering the potential perioperative risk based on impaired renal and hepatic function and chronic heart failure, as well as the patient's request, our strategy was to perform EVAR to reduce preload and control of the shunt volume. Additional open repair, such as fistula closure and graft replacement of the abdominal aortic aorta, could be conducted if EVAR could not manage the ACF.

**Fig. 2:**
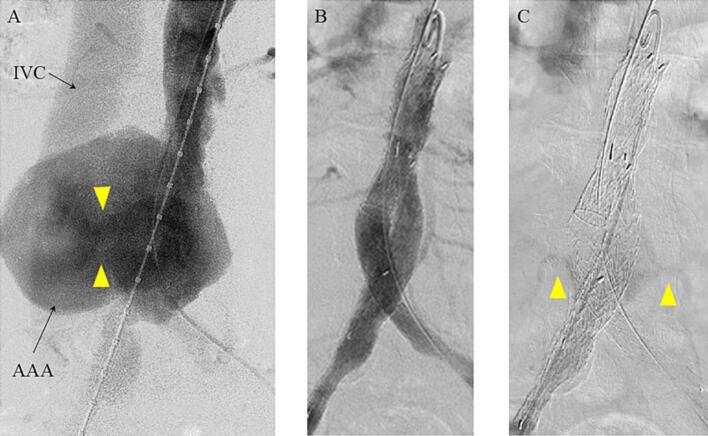
Intraoperative Angiography. A) Initial aortogram demonstrated abdominal aortic aneurysm and inferior vena cava was contrasted in the arterial phase through a huge aortocaval fistula (shown as yellow arrows). (B, C) Final aortogram revealed no Type I/III endoleak in the early phase (B) and a Type II endoleak from the third lumbar arteries in the late phase (C) (shown as yellow arrows). Abbreviations: AAA, abdominal aortic aneurysm; IVC, inferior vena cava. (For interpretation of the references to colour in this figure legend, the reader is referred to the web version of this article.)

**Fig. 3:**
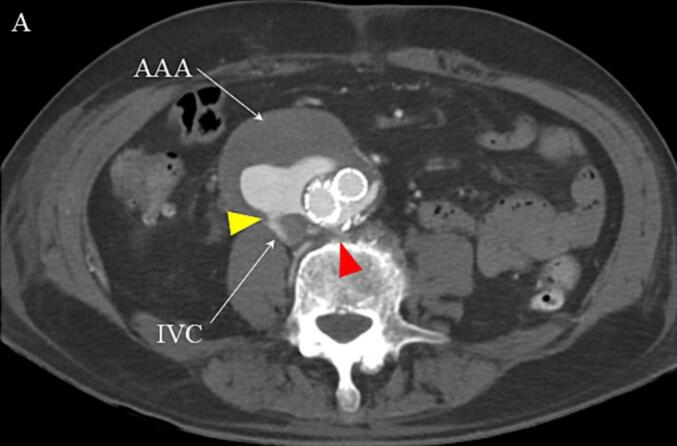
Postoperative Computed Tomography on the Postoperative Day 2. A residual Type II endoleak from the lumbar artery (shown as a red arrow) and reduced aortocaval fistula (shown as a yellow arrow). Abbreviations: AAA, abdominal aortic aneurysm; IVC, inferior vena cava. (For interpretation of the references to colour in this figure legend, the reader is referred to the web version of this article.)

The procedure was performed under general anaesthesia. Both common femoral arteries were exposed. After puncturing both common femoral arteries, aortography was performed to detect the location of the ACF (Fig. 2A). The main device, a Gore Excluder C3 (RLT231412J; W. L. Gore & Associates, Flagstaff, AZ, USA), was deployed just below the left renal artery, and the Gore Excluder contralateral leg (PLC121200J) was deployed from the contralateral leg to the distal side of the left common iliac artery. Intraoperative aortography revealed no Type I or Type III endoleak (EL) in the early phase (Fig. 2B) but revealed Type II EL originating from the bilateral lumbar arteries and flowing into the aortic aneurysm during the venous phase (Fig. 2C). Although a slight contrast agent flow from the aortic aneurysm into the IVC persisted, the shunt volume was significantly reduced. The patient's urinary output increased the day after EVAR, with dyspnoea and oedema improving several days later. Postoperative laboratory values demonstrated an improvement in renal function (creatinine, 1.45 mg/dL) and decreased cardiac marker levels (brain natriuretic peptide, 117 pg/mL). Postoperative contrast-enhanced CT showed the persistence of a slight Type II EL and minimal shunting into the IVC (Fig. 3). However, the AAA diameter decreased to 50.4 mm × 72.4 mm. The patient recovered uneventfully and was discharged on postoperative day 11.

## 3 Discussion

ACF is a relatively rare comorbidity of AAA, accounting for 3–4 % of all cases of ruptured aortic aneurysms [7]. The IVC is the predilection site for ACF. The various symptoms associated with ACF include heart failure, renal failure, venous thrombosis, pulmonary embolism, and pulmonary hypertension [1]. The presence of multiple symptoms often complicates preoperative diagnosis, contributing to a high mortality rate of 22–50 % [1,2,8]. Mortality is particularly elevated, and intra- and postoperative managements becomes especially challenging when patients present in a state of shock preoperatively [3].

Although the standard surgical intervention for AAA complicated by ACF is abdominal aortic graft replacement and fistula closure, serious perioperative concerns should be considered. These concerns include general anaesthesia risks, bleeding management, and intraoperative pulmonary oedema, particularly due to hemodynamic changes following a rapid decrease in shunt volume [1,2,8]. Recent reports have demonstrated the feasibility and good surgical outcomes of EVAR for patients with AAA complicated by ACF [4,5]. Conversely, complete fistula closure via EVAR remains challenging in AAA cases with ACF, though it may be feasible for patients with common iliac artery aneurysm with ACF [9]. One of the mechanisms may involve the inflow pressure into the aortic aneurysm from the lumbar and inferior mesenteric arteries being lower than the aortic pressure, a factor reported to reduce the risk of re-rupture. Additionally, a second mechanism underlying the favourable surgical outcomes of EVAR may involve the fistula to the IVC acting as an escape route for blood flow from the aneurysm, resulting in shrinkage of the aortic aneurysm [5]. Although a more in-depth analysis is necessary to fully assess the feasibility of EVAR for patients with aortic aneurysms complicated by ACF, one report demonstrated that EVAR was prioritised as a life-saving procedure in high-risk patients with full understanding of the possibility of a residual arteriovenous shunt. In such cases, additional interventions could be performed if the patient's condition stabilised and the aneurysm enlarged [8]. Nevertheless, no consensus currently exists on whether fistula closure should be performed in low-risk patients [10].

In this high-risk patient with comorbid chronic heart failure and renal dysfunction, we opted to perform EVAR not for the complete ACF closure, but to prevent rupture, reduce preload, and control the shunt volume. This approach resulted in improvement of the patient's condition and shrinkage of the AAA diameter. Collectively, this case provides valuable guidance on surgical strategies for high-risk patients with AAA complicated by ACF.

## 4 Conclusion

This case provides insights into the successful emergency EVAR treatment for high-risk patients with chronic heart failure and renal dysfunction secondary to increased preload from an ACF, highlighting the importance of EVAR as an alternative treatment for patients with difficult surgical situations or those who refuse open surgery.

## Consent

Written informed consent was obtained from the patient for publication of this case report and accompanying images. Identifying details have been omitted. A copy of the written consent is available for review by the Editor-in-Chief of this journal on request.

## Ethical approval

Ethical approval was not required for this case report in our institution.

## Funding

No funding.

## Author contribution

Dr. Kosuke Tsumura — collecting the data, writing the article, reviewing patient notes, writing articles, analyzing images, and approving the final submission.

Dr. Masateru Uchiyama — corresponding author; collecting the data, writing the article, reviewing patient notes, writing articles, analyzing images, and approving the final submission.

## Guarantor

Dr. Masateru Uchiyama.

## Research registration number

N/A.

## Conflict of interest statement

The authors declare no financial, personal, or other conflicts of interest that could induce bias.
